# Starch phosphorylation associated SNPs found by genome-wide association studies in the potato (*Solanum tuberosum* L.)

**DOI:** 10.1186/s12863-019-0729-9

**Published:** 2019-03-18

**Authors:** Vadim K. Khlestkin, Irina V. Rozanova, Vadim M. Efimov, Elena K. Khlestkina

**Affiliations:** 1 Institute of Cytology and Genetics, Siberian Branch of the Russian Academy of Sciences, Lavrentjeva Ave. 10, Novosibirsk, 630090 Russia; 2 Russian Research Institute of Farm Animal Genetics and Breeding - Branch of the L.K. Ernst Federal Science Center for Animal Husbandry, St. Peterburg-Tyarlevo, Moskovskoe shosse, 55a, 196625 Russia; 3 Novosibirsk State University, Pirogova Str., 1, Novosibirsk, 630090 Russia; 4 N.I. Vavilov All-Russian Research Institute of Plant Genetic Resources (VIR), Bolshaya Morskaya Str., 42-44, St. Petersburg, 190000 Russia

## Abstract

**Background:**

The natural variation of starch phosphate content in potatoes has been previously reported. It is known that, in contrast to raw starch, commercially phosphorylated starch is more stable at high temperatures and shear rates and has higher water capacity. The genetic improvement of phosphate content in potato starch by selection or engineering would allow the production of phosphorylated starch in a natural, environmentally friendly way without chemicals. The aim of the current research is to identify genomic SNPs associated with starch phosphorylation by carrying out a genome-wide association study in potatoes.

**Results:**

A total of 90 *S. tuberosum* L. varieties were used for phenotyping and genotyping. The phosphorus content of starch in 90 potato cultivars was measured and then statistically analysed. Principal component analysis (PCA) revealed that the third and eighth principal components appeared to be sensitive to variation in phosphorus content (*p* = 0.0005 and *p* = 0.002, respectively). PC3 showed the correlation of starch phosphorus content with allelic variations responsible for higher phosphorylation levels, found in four varieties. Similarly, PC8 indicated that hybrid 785/8–5 carried an allele associated with high phosphorus content, while the Impala and Red Scarlet varieties carried alleles for low phosphorus content. Genotyping was carried out using an Illumina 22 K SNP potato array. A total of 15,214 scorable SNPs (71.7% success rate) was revealed. GWAS mapping plots were obtained using TASSEL based on several statistical models, including general linear models (GLMs), with and without accounting for population structure, as well as MLM. A total of 17 significant SNPs was identified for phosphorus content in potato starch, 14 of which are assigned to 8 genomic regions on chromosomes 1, 4, 5, 7, 8, 10, and 11. Most of the SNPs identified belong to protein coding regions; however, their allelic variation was not associated with changes in protein structure or function.

**Conclusions:**

A total of 8 novel genomic regions possibly associated with starch phosphorylation on potato chromosomes 1, 4, 5, 7, 8, 10, and 11 was revealed. Further validation of the SNPs identified and the analysis of the surrounding genomic regions for candidate genes will allow better understanding of starch phosphorylation biochemistry. The most indicative SNPs may be useful for developing diagnostic markers to accelerate the breeding of potatoes with predetermined levels of starch phosphorylation.

**Electronic supplementary material:**

The online version of this article (10.1186/s12863-019-0729-9) contains supplementary material, which is available to authorized users.

## Background

The potato is the third most important food crop in the world, and it has reliably held this position for centuries [1]. The plant was brought from Peru and Chile to Europe around the year 1570 [2] and reached Russia in the late seventeenth century [3]. The potato was used almost exclusively for food purposes in Russia until the very end of the nineteenth century, when potato starch became a standalone product. Since that time, potato starch has been used in both the food and technical industries. In the food industry, the potato is a common filler, gelling and texturizing agent, as well as a precursor for glucose, molasses, and dextrin production. Technical applications for starch are mainly in textile and paper manufacturing. Despite the fact that Russia is the world’s third largest manufacturer of potatoes [4], it mainly imports potato starch due to the limited number of potatoes processed into raw starch. A negligible amount of the raw starch is further transformed into chemical and biochemical products [5]. Considering that potato tubers cannot be stored for long periods of time, the use of potatoes in the form of starch and products from starch modification is a good alternative. To increase the appeal of potato starch as a feedstock for various industries, substantial attempts to improve its molecular composition and physical and chemical properties are being made. The manipulation of certain gene networks may result in modified potato lines that produce starch with particular properties. This process has already been used to develop potato varieties with altered amylose and amylopectin content to produce starch that yields a clearer and more viscose gel, stable in freeze-thaw cycles [6].

Another feature of potato starch that could be improved with significant practical benefits is its phosphate content. Phosphate groups in potato starch are normally chemically bound to amylopectin molecules with approximately one phosphate group per 200–300 glucose units (0.05–0.08% P). The negatively charged phosphate groups result in better dispersion of the polysaccharide chains in water due to mutual Coulomb repulsion. In industry, phosphorylated starch is manufactured in a chemical process that results in the addition of 1–3% phosphate content by mass. Commercially phosphorylated starch is known as food additive E1410 and functions as a texturizing and stabilizing agent. In contrast to raw starch, it is chemically more stable at high temperatures and shear rates and has a higher water capacity. It has been demonstrated that the physical and chemical properties of potato starch gels is dependent on its phosphate content [7]. Thus, genetically improving the phosphate content in potato starch through selection or genetic engineering would allow the production of phosphorylated starch in a natural, environmentally friendly way without the use of chemicals or other pollutants. Additionally, phosphorus is important for proper nutrition, and potatoes and starch high in phosphate would be valuable as food.

There have been some successful attempts to genetically modify potatoes that produce starch with a higher phosphate content. The expression of the laforin protein in the tuber [8] resulted in an average increase of 19% in phosphate content with a simultaneously increase in the amount of amylopectin in the starch. A potato α-glucan, water dikinase (GWD1) introduced into tubers of the amylose-containing line Kardal and the amylose-free mutant *amf* resulted in two contrasting effects; some plants showed higher phosphate content than the corresponding control, while others exhibited lower phosphate content, thereby generating starches with broad-scale variation in phosphate content [9]. The introduction of an (engineered) 4, 6-α-glucanotransferase (GTFB) from *Lactobacillus reuteri* 121 into the same lines resulted in a significant increase in starch phosphate content in the *amf* line, while starches from the Kardal background did not show any changes in phosphate content [8].

It was shown that α-glucan, water dikinase (GWD/GWD1) and phosphoglucan, water dikinase (PWD/GWD3) enzymes play key roles in starch metabolism. Improved physical and chemical properties of starches isolated from GWD-deficient plants were described using *Arabidopsis* as a model [10].

In addition to reverse genetic approaches, the tools of forward genetics such as QTL analysis [11, 12] and association mapping [13] were used to reveal loci associated with starch phosphorylation in potatoes. The development of the 22 K SNP potato array with a high density of markers (on average, one SNP per 40 kbp compared to one marker per 4 Mbp in the abovementioned studies) allows us the opportunity to broaden the number of identified genomic loci associated with starch phosphorylation.

We performed a genome-wide association study using a 22 K SNP potato array to find novel genomic regions associated with starch phosphorylation.

## Methods

### Plant material

The set of 90 potato (*Solanum tuberosum* L.) varieties from ICG “GenAgro” collection (Novosibirsk, Russia) was grown during the period May to October 2017 in the same field in Novosibirsk region (Michurinsky settlement, 54°52′ N and 83°00′ E). A subset of 8 varieties was preliminary grown during the period May to October 2016 in the same field. Growing of potato plants was performed according to the standard procedure. Briefly, seed tubers of all cultivars were planted in two rows with 0.75 m spacing and 0.3 m distance between the plants on the rows. In total, 10 plants were planted in the row, so, the length of each row was 10 m. Each cultivar was planted in three replicates, and distances between the replicates’ plots were 2 m. Sowing was performed in the first decade of May and harvesting in the 3rd decade of September.

After harvesting tubers were stored for 3 weeks at + 4 °C. Only healthy tubers were collected for further analyses. From the healthy tubers, 25% of the smallest and largest tubers were removed. Among the rest, only five morphologically typical for a given variety tubers were selected for starch isolation.

### Starch isolation

Potato starch was isolated form the tuber according to the typical procedure, described elsewhere (for example, see [14].

### DNA isolation and genotyping

DNA was isolated from tubers skin using DNeasyPlant Mini Kit (Qiagen) according to the standard procedure. Concentration and purity of the isolated DNA were tested by gel electrophoresis and micro spectrophotometry on Nanodrop 2000 equipment.

All 90 accessions were genotyped using Illumina 22 K SNP potato array (GGP Potato V3) at the Traitgenetics GmbH (Gatersleben, Germany). The Illumina Infinium procedure was performed according to the manufacturer’s protocol. None of the samples were failed for the analysis.

### Phosphorus content analysis

Analysis of the starch samples was performed using phosphomolibdate method with spectrophotometric detection according to the standard procedure, described in GOST 7698–93 “Starch. Rules for acceptance and methods of analysis” (Russian and Belarus standard, correlates with ISO 3946-82). In brief, starch probe was decomposed in a mixture of sulfuric and nitric acids, neutralized and reacted with ammonium molibdate. Spectrophotometric detection at 825 nm allowed to determine content of phosphorus in the initial starch probe with the help of the calibration curve.

### PCA and population structure analysis

The Principal component analysis (PCA) was considered with genotyping data calculated through the distance matrix. It was done using software packages STATISTICA 8 [15]. In order to calculate the population structure matrix (Q-matrix) containing membership coefficients for each individual of potato mapping panel the genotyping data was analyzed by Bayesian cluster analysis in STRUCTURE v.2.3.4 [16]. The number of population was taken as k = 3, which corresponded to number of clusters according to STATISTICA.

### Association analysis

Different statistical models were tested on disease resistance scores (separately for each of four isolates) with the help of TASSEL 5 package [17] to detect significant marker associations: (1) general linear model (GLM) without taking into account population structure, (2) GLM with using a Q-matrix of population membership (GLM) taking into account the population structure, (3) GLM with taking into account population membership estimates derived from principal components analysis (GLM + PCA), and (4) a composite approach that combines both Q-matrix and the average relationship between individuals or lines (null matrix) represented in TASSEL as a linear model (MLM).

Since TASSEL have specially been developed for diploid genome analysis, we re-coded tetraploid potato genome from four-letter code to numerical, taking into account the dose of certain allele. After the re-coding, 0 was assigned to effector allele and 1 – as non-effector allele, and their intermediate forms were coded as 0.75, 0.5 and 0.25. For example, AAAA allele is reflected as 1, AAAG – as 0.75, AAGG – as 0.5, AGGG – as 0.25, and GGGG – as 0.

To identify significant SNPs two corrections were used: (i) the Bonferroni correction, where the significant threshold (0.05) is divided by the total number of tests, in this case, the total number of markers (27,319), giving threshold 1.8302*10^− 6^, and (ii) the false discovered rate (FDR) that was calculated for each isolate in each model. Percentage of random was < 10%.

## Results and discussion

### Phenotyping, genotyping and population structure

**Fig. 1:**
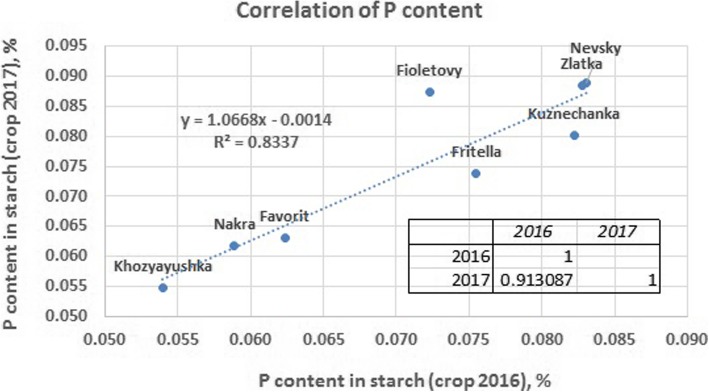
A strong positive correlation of starch phosphorylation in the tubers of potato varieties harvested in 2016 and 2017

Initially, the starch phosphorus content was determined for 8 varieties of potato harvested in 2016 and 2017. A strong positive correlation of this trait between 2 years (Pearson’s coefficient 0.91) was found in most of the cultivars (Fig. 1). The correlation of high phosphorus content in the crops for both years indicated that genetics rather than the environment contributed to the variation among the genotypes for starch phosphorylation level. Thus, to perform the association mapping, the starch phosphorus content was determined for 90 varieties of potato harvested in 2017.

The SNP-genotyping of the 90 varieties harvested in 2017 was performed on an Illumina 22 K potato array (GGP Potato V3) and revealed a set of 15, 214 (71.7%) scorable SNPs. SNPs were treated as significant only if they were discovered in > 95% of the studied genotypes. We used the chromosomal position of the SNPs [18] to arrange the marker dataset on the corresponding chromosomes with the help of a specially written script. The average coverage was one SNP per 40 kbp calculated from the known length of the potato genome (n), which is approximately 840 Mbp [19]. Previous association studies and QTL analyses for phosphorus content in potato tubers were based on a restricted number of markers with an average of 1 marker per 4 Mbp [11–13].

**Fig. 2:**
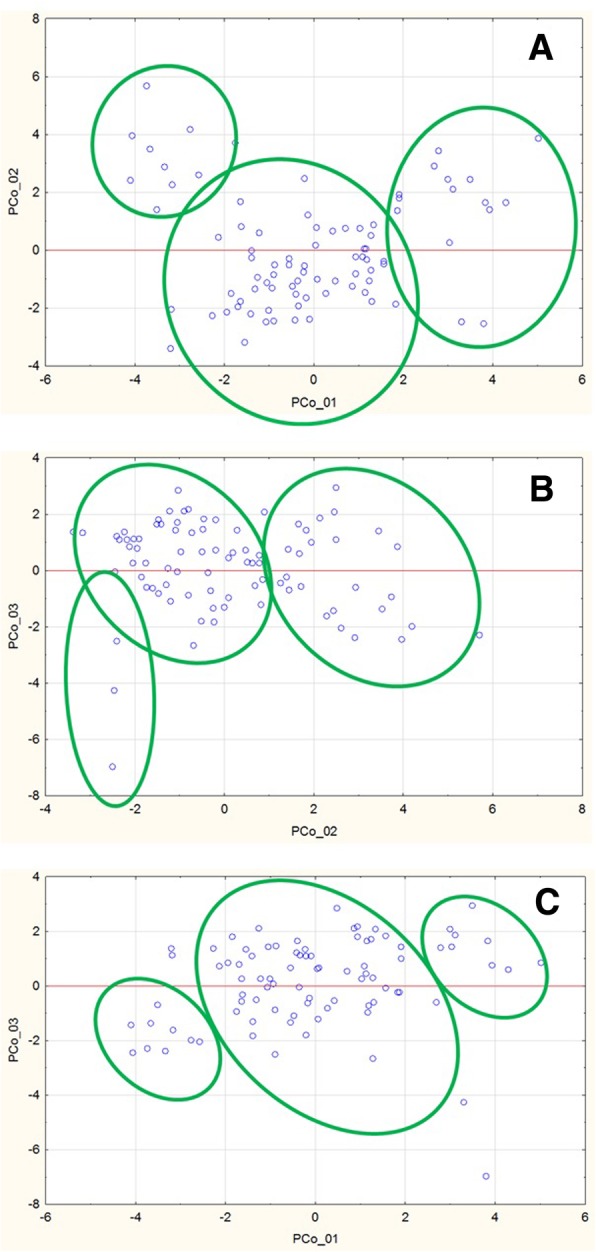
Three clusters of the potato population studied based on SNP-genotyping data. **a** Principal components 1–2; **b** principal components 2–3; **c** principal components 1–3

Population structure analysis showed that for the set of 90 potato varieties, a total of 23 principal components contributed more than 1% of common genotype variance. The first three components described 31.8% of variance. Two dimensional projections of the first three principal components using PC1-PC2, PC2-PC3 and PC1-PC3 (Fig. 2) uncovered three clusters, highlighted in green ellipses. The projection of PC1–3 also revealed that there are extreme differences between some varieties in the study set.

**Fig. 3:**
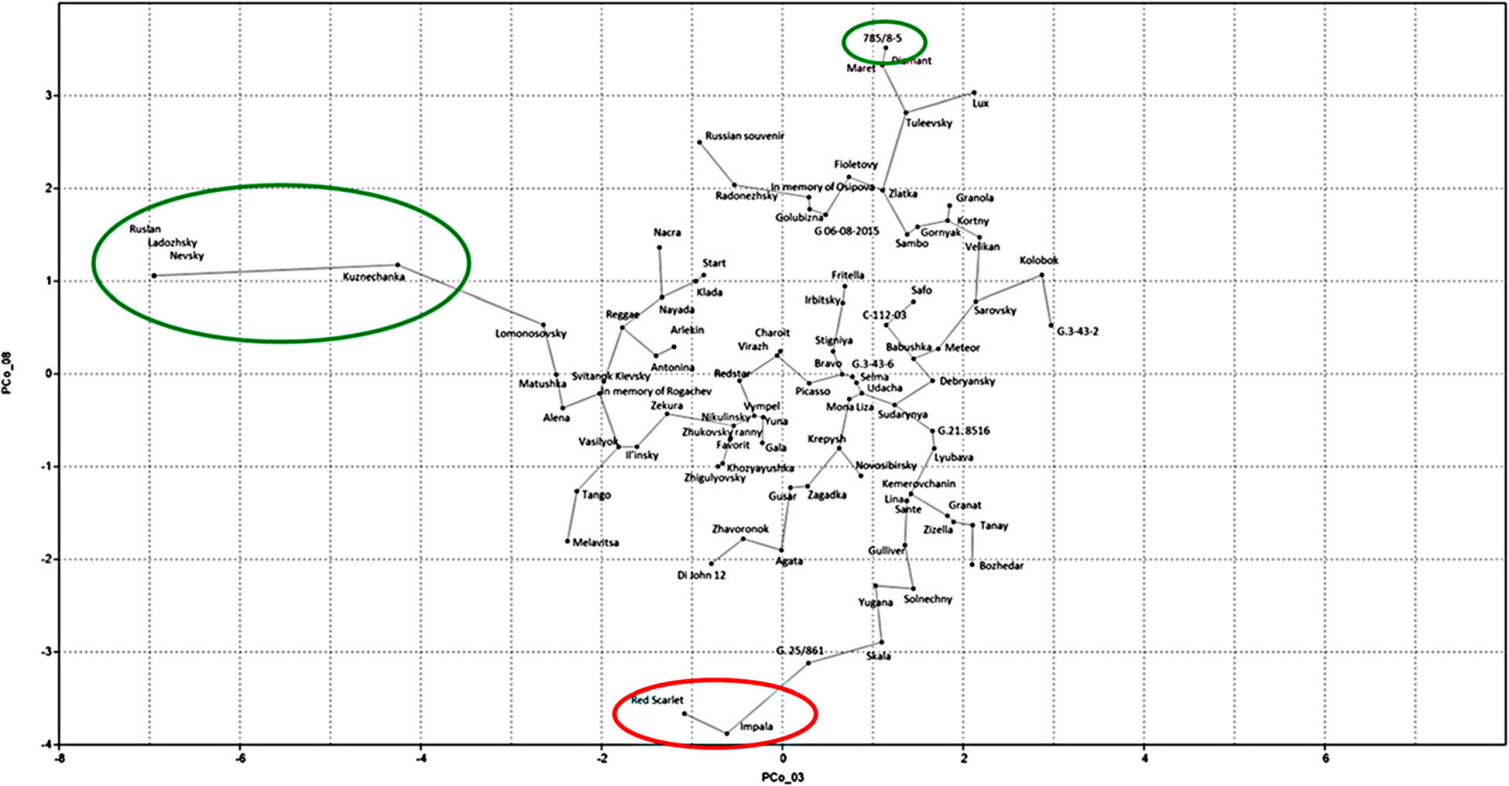
Correlation of the third and eighth principal components. Varieties with high and low phosphorous content are marked with green and red ellipses, respectively

Only the third and eighth principal components (*p* = 0.0005 and *p* = 0.002, respectively) appeared to account for phosphorus content. Correlation of the starch phosphorus content with PC3 suggested that the Ladozhsky, Nevsky, Ruslan and Kuznechanka varieties carry alleles responsible for higher phosphorus content (Additional file 1). Similarly, the correlation with PC8 suggested variety 785/8–5 carried an allele pattern associated with high phosphorus content, and the Impala and Red Scarlet varieties carried alleles for low phosphorus content (Additional file 1). Figure 3 reflects the correlations in the third and eighth principal components. Varieties with high and low phosphorous content are marked with green and red ellipses, respectively.

### Association mapping

The association-mapping plots were obtained with the TASSEL program by using the SNP-genotyping and phenotyping results for all 90 varieties. QQ- and Manhattan plots shown in Additional files 2 and 3, respectively, were obtained from the application of different statistical methods to the genotyping and phenotyping correlation results. The basic approach used GLM without considering population structure. To build on this, GLM was used with a Q-matrix of population membership, which takes into account population structure. Population membership estimates serve as covariates in the model and can be derived using principal component analysis (PCA) [20]. For each marker-trait combination, GLM finds the ordinary least squares solution as described previously [21]. The model can include main effects, interactions, nested effects and covariates. A composite approach that combines both the Q-matrix and the average relationship between individuals or lines (null matrix) represented in TASSEL as a linear model (MLM) function has been shown to be superior [22] to GLM methods. Finally, we used all 4 approaches: (1) general linear model (GLM) without considering population structure, (2) GLM with a Q-matrix of population membership (GLM) taking into account population structure, (3) GLM taking into account population membership estimates derived from principal components analysis (GLM + PCA), and (4) a composite approach that combines both the Q-matrix and the average relationship between individuals or lines (null matrix) represented in TASSEL as a linear model (MLM) (Additional file 3). A total of 17 significant possible SNPs was found (Table 1). SNPs with a *p*-value that overcame either 3.29 e^− 6^ (Bonferroni level) or the less strict FDR (false discovery rate) criteria were considered. The Bonferroni correction sets very strict criteria; overcoming it implies the greatest likelihood of SNP-trait association, but permits false negative results, omitting some trait-associated SNPs [23]. To counteract this, the less strict FDR criteria were also applied. Three of the 17 SNPs identified were not assigned to a chromosome (Table 1). The rest of the 14 markers were assigned to chromosomes 1, 4, 5, 7, 8, 10 and 11. Twelve of these 14 SNPs are located in protein-coding regions (Table 2).

**Table 1:** Significant SNPs discovered with the four statistical models

No	Marker	Chromosome	Position	Correction	*p*-value	Minor allele frequency	Polymorphism	Allele associated with high phosphorylation level
1	PotVar0043426^a^	1	69,895,805	Bonferroni	0,00000064	0,35	A/G	G
2	solcap_snp_c2_17529^a^	1	69,902,011	Bonferroni	0,00000131	0,32	A/G	G
3	solcap_snp_c1_5759^a^	1	69,917,893	Bonferroni	0,00000167	0,40	A/G	G
4	solcap_snp_c2_17530^a^	1	69,918,804	Bonferroni	0,00000104	0,33	T/A	A
5	PotVar0043516^a^	1	69,872,835	Bonferroni	0,00000303	0,38	A/G	G
6	solcap_snp_c1_2519^c^	1	79,487,269	Bonferroni	0,00000332	0,21	T/C	C
7	PotVar0084444^d^	4	58,263,204	FDR	0,0000887	0,04	T/C	C
8	PotVar0018043^b^	5	33,966,509	FDR	0,0000165	0,24	A/G	G
9	solcap_snp_c2_50231^b^	5	36,834,630	FDR	0,000027	0,33	T/C	T
10	solcap_snp_c2_38828^b^	7	588,137	FDR	0,0000375	0,21	A/G	G
11	solcap_snp_c1_6252^d^	8	20,465,382	FDR	0,0000661	0,05	A/G	A
12	PotVar0065847^b^	10	134,951	FDR	0,0000245	0,13	A/G	G
13	PotVar0065745^b^	10	197,180	FDR	0,0000339	0,13	A/G	G
14	solcap_snp_c1_2187^b^	11	2,562,328	FDR	0,000036	0,46	A/G	G
15	solcap_snp_c2_55899^c^	Unknown	Unknown	FDR	0,00000901	0,30	T/G	T
16	solcap_snp_c2_53177^d^	Unknown	Unknown	FDR	0,0000373	0,05	T/C	T
17	solcap_snp_c2_7785^d^	Unknown	Unknown	FDR	0,0000663	0,07	A/G	G

^a^GLM, ^b^GLM + Q, ^c^GLM + PCA, ^d^MLM

**Table 2:** Description of genes and their products (according Plant.Essemble.com), associated with the protein-coding SNPs identified in the current study

SNP-marker (nucleotide substitution)	Chr	Gene code	Gene statistics	Transcript name	Protein statistics
PotVar0043516 (syn)	1	PGSC0003DMG400026032	Exons: 7, Coding exons: 7, Transcript length: 1938 bps, Translation length: 645 residues	Carotenoid cleavage dioxygenase 7	Ave. residue weight: 112.975 g/mol Charge: 5.0 Isoelectric point: 7.0484 Molecular weight: 72,869.07 g/mol Number of residues: 645 aa
PotVar0043426 (syn) solcap_snp_c2_17529 (syn)	1	PGSC0003DMG400026030	Exons: 20, Coding exons: 20, Transcript length: 3574 bps, Translation length: 760 residues	Mitochondrial elongation factor	Ave. residue weight: 110.614 g/mol Charge: 1.0 Isoelectric point: 6.6146 Molecular weight: 84,066.49 g/mol Number of residues: 760 aa
solcap_snp_c2_17530 (syn) solcap_snp_c1_5759 (syn)	1	PGSC0003DMG400026029	Exons: 7, Coding exons: 7, Transcript length: 1297 bps, Translation length: 332 residues	Malate dehydrogenase	Ave. residue weight: 109.454 g/mol Charge: 15.5 Isoelectric point: 9.8591 Molecular weight: 36,995.61 g/mol Number of residues: 338 aa
solcap_snp_c1_2519 (Val < => Ile)	1	PGSC0003DMG402018257	Exons: 3, Coding exons: 3 Transcript length: 3145 bps Translation length: 386 residues	Leucine-rich repeat-containing protein	Ave. residue weight: 114.581 g/mol Charge: − 0.5 Isoelectric point: 6.4430 Molecular weight: 62,103.08 g/mol Number of residues: 542 aa
PotVar0084444 (syn)	4	PGSC0003DMG400024812	Exons: 10, Coding exons: 9, Transcript length: 1841 bps, Translation length: 408 residues	Maltose transporter	Ave. residue weight: 111.048 g/mol Charge: 17.5 Isoelectric point: 9.9764 Molecular weight: 45,307.70 g/mol Number of residues: 408 aa
solcap_snp_c2_38828 (syn)	7	PGSC0003DMG400011132	Exons: 9, Coding exons: 9, Transcript length: 1768 bps, Translation length: 496 residues	NADP-dependent glyceraldehyde-3-phosphate dehydrogenase	Ave. residue weight: 107.120 g/mol Charge: 3.5 Isoelectric point: 7.1101 Molecular weight: 53,131.55 g/mol Number of residues: 496 aa
solcap_snp_c1_6252 (syn)	8	PGSC0003DMG400029895	Exons: 11, Coding exons: 10, Transcript length: 2175 bps, Translation length: 529 residues	Importin alpha	Ave. residue weight: 110.400 g/mol Charge: − 11.5 Isoelectric point: 4.9041 Molecular weight: 58,401.63 g/mol Number of residues: 529 aa
PotVar0065745 (syn)	10	PGSC0003DMG401011292	Exons: 4, Coding exons: 4, Transcript length: 1128 bps, Translation length: 375 residues	Strong similarity to naringenin 3-dioxygenase	Ave. residue weight: 112.754 g/mol Charge: − 20.5 Isoelectric point: 4.3935 Molecular weight: 42,282.69 g/mol Number of residues: 375 aa
PotVar0065847 (syn)	10	PGSC0003DMG400011295	Exons: 6, Coding exons: 6, Transcript length: 957 bps, Translation length: 180 residues	Thylakoid membrane phosphoprotein 14 kDa, chloroplastic	Ave. residue weight: 105.760 g/mol Charge: 1.0 Isoelectric point: 7.7746 Molecular weight: 19,036.87 g/mol Number of residues: 180 aa
solcap_snp_c1_2187	11	PGSC0003DMG400016217	Exons: 1, Coding exons: 1, Transcript length: 1766 bps, Translation length: 393 residues	Ring finger protein	Ave. residue weight: 113.742 g/mol Charge: − 8.5 Isoelectric point: 5.1056 Molecular weight: 44,700.59 g/mol Number of residues: 393 aa

*Syn* synonymous, *Val* valine, *Ile* isoleucine

### Chromosome 1

Six significant SNPs were found on chromosome 1 (Table 1). Five of the six markers were mapped closely to each other, corresponding to one genomic region approximately 50 kbp in length (between 69,872,835 and 69,918,804 bp of chromosome 1). The SNPs were located in protein-coding regions of genes: 1 SNP was in a gene encoding carotenoid cleavage dioxygenase 7; 2 SNPs were located in a gene encoding a mitochondrial elongation factor; and 2 SNPs were in a malate dehydrogenase gene (Table 2). Allelic variation in these SNP loci is to due synonymous substitutions only.

Another significant SNP was located about 10 Mbp from the genomic region described above (Table 1). The SNP was found in the gene PGSC0003DMG402018257 coding for a leucine-rich, repeat-containing protein (Table 2). Allelic variation in this SNP locus results in an amino acid substitution, Val < => Ile, though does not change the main properties of the amino acid residue, since both valine and isoleucine are aliphatic hydrophobic amino acids. An association between potato chromosome 1 and the phosphorus content of potato starch has never before been reported. Further investigation of the two genomic regions identified on chromosome 1 is needed to find and validate candidate genes associated with phosphorus content variation in potato tubers.

### Chromosome 4

A single significant SNP was revealed on chromosome 4 (Table 1). The SNP was in a gene encoding a maltose transporter. Allelic variation in this SNP locus does not result in an amino acid change (Table 2). No QTL has previously been mapped to chromosome 4 for phosphorus content. However, Carpenter et al. [13] reported a relationship between the starch branching enzyme I gene (*SBEI*) and starch phosphorylation. The *SBEI* location is 4:71586223–71,597,347, which is approximately 13 Mbp distal from the PotVar0084444 SNP, suggesting that the phosphorus content-associated region identified on chromosome 4 in the current study is not related to *SBEI*.

### Chromosome 5

Two significant SNPs were found on chromosome 5 (Table 1). These SNPs were found in the non-coding regions of the chromosome. They are approximately 2.8 Mbp from each other. Previously, a QTL for phosphorus content related to potato starch was reported for this locus [11, 12]. Werij et al. [12] suggested that this locus was related to the *GWD* (*GWD1*) gene encoding α-glucan, water dikinase (syn.: Starch-granule-bound R1 protein). Carpenter et al. [13] also observed an association between the *GWD* gene and the phosphorus content in tuber starch. The *GWD* gene location is 5:9823451–9,838,970. The distance between this gene and the region found in the current study is greater than 30 Mbp.

### Chromosome 7

Potato chromosome 7 has never before been reported to be associated with phosphorus content in tuber starch. Here, the significant chromosomal region was found to be associated with the solcap_snp_c2_38828 marker (Table 1). The SNP was located in the gene PGSC0003DMG400011132 encoding a NADP-dependent glyceraldehyde-3-phosphate dehydrogenase protein. Allelic variation in this SNP locus does not result in an amino acid substitution (Table 2). Further investigation of this gene, comparisons of allelic differences in coding regions between varieties with high and low phosphorus content as well as application of reverse genetics tools are needed to check whether this gene contributes to phosphorylation levels in potato tuber starch.

### Chromosome 8

The association between potato chromosome 8 and starch phosphorylation has not been reported previously. A significant SNP was found on chromosome 8 (Table 1). The SNP was located in the gene coding region for an importin alpha protein. Nucleotide changes in this SNP locus are synonymous (Table 2). The product of this gene is responsible for alpha protein import – a large class of proteins, which may include enzymes responsible for phosphorylation. Without detailed experimental investigation of this gene and neighbouring genes in the chromosomal region identified in the current study, it is not possible to assume a candidate gene.

### Chromosome 10

Potato chromosome 10 has never been reported to be associated with phosphorus content in tuber starch before. Two significant SNPs were revealed in the current study (Table 1). These SNPs were in protein-coding genes, one having strong similarity to naringenin 3-dioxygenase (PGSC0003DMG401011292) and the other to thylakoid membrane phosphoprotein (PGSC0003DMG400011295). They are situated approximately 63 kbp from each other. Naringenin 3-dioxygenase participates in biosynthesis of secondary phenolic metabolites and is most likely unrelated to starch phosphorylation. Thus, significance of the SNP in PGSC0003DMG401011292 may be explained by its close linkage to PGSC0003DMG400011295 or other genes affecting phosphorus content. Substitution in the SNP found in the gene PGSC0003DMG400011295 is synonymous. Further investigation of this gene and neighbouring genes is needed.

### Chromosome 11

A single significant SNP was located on chromosome 11 (Table 1). It was found in a gene encoding a ring finger protein. Allelic variation in this SNP locus does not result in an amino acid change (Table 2). This is the first report of a region associated with starch phosphorylation on chromosome 11 and is of some interest; however, without further investigation of this region, it is difficult to make any conclusions about possible candidate genes.

In addition to the listed potato chromosomes above, the 2nd and 9th chromosomes are associated with tuber starch phosphorylation [11–13]. By sequencing amplicons and comparing this with phenotypic data, putative candidate genes involved in starch metabolism were genotyped [13], and markers associated with C6-phosphorylation (*GWD* gene, chromosome 5), C3-phosphorylation (*PWD/GWD3* gene, chromosome 9), and phosphorylation in both positions (*SBEI* - chromosome 4, *SBEII* - 9, *SSII* - 2, and *SSIII* - 2) were uncovered (chromosome assignment of the genes is given according to Plant.Essemble.com or taken from a review [6]). Carreno-Quintero, et al. [11] revealed QTLs on chromosomes 2, 5, and 9 and reported the correlation of starch phosphorylation with seven metabolites (β-Ala, GABA, L-Asp, Ala, butanoic acid, and two unknown compounds). QTLs for these metabolites colocalize with at least one of the starch phosphorylation QTLs. Starch phosphorylation QTLs were found and mapped on chromosomes 2, 5 and 9 by Werij et al. [12]. These QTLs colocalize with *SSII* on chromosome 2, *GWD* on chromosome 5, and *StPho2* on chromosome 9 [12].

## Conclusions

A genome-wide association study using a 22 K SNP potato array allowed 8 novel genomic regions on chromosomes 1, 4, 5, 7, 8, 10, and 11 associated with starch phosphorylation to be found. Some of the SNPs identified were located in non-coding genomic regions. Allelic variation in the SNPs found in protein coding regions was not related to changes in protein structure or function. Further validation of the SNPs identified and the analysis of the surrounding genomic regions for candidate genes will allow a better understanding of starch phosphorylation biochemistry. Among the genes carrying significant SNPs identified in the current study, the gene on chromosome 7 encoding a NADP-dependent glyceraldehyde-3-phosphate dehydrogenase protein is a primary target for a reverse genetic investigation of a potential functional association with tuber starch phosphorus content.

## Additional files

Additional file 1: Scatterplots of phosphorus content in starch of potato varieties analyzed, showing sensitivity of the third (A) and eighth (B) components to phosphorus content. The blue ellipses show varieties, which high or low phosphorous contents in starch are genetically determined. (PDF 314 kb)

Additional file 2: QQ-plots (quantile-quantile plots) for the models: (1) GLM without correction for population structure; (2) GLM + Q: GLM + Q-matrix to account for population structure; (3) GLM + PCA, (4) MLM. (PDF 282 kb)

Additional file 3: Association mapping results for phosphorous content scorings in potato starch using different models: GLM without accounting for population structure, GLM + Q (GLM + Q-matrix to account for population structure), GLM + PCA, and MLM. Dash line named “Bonferroni” corresponds the Bonferroni threshold. Dash line named “FDR” corresponds the FDR (false discovered rate) threshold. Vertical axes show the –log (10) of marker trait association *p*-value. Chr1–12 – chromosome assignment of loci. Chr0 – unassigned loci. (PDF 787 kb)
