# Supplementary material for: Starch phosphorylation associated SNPs found by genome-wide association studies in the potato (Solanum tuberosum L.)
Source: BMC Genet. 2019 Mar 18;20(Suppl 1):29. doi: 10.1186/s12863-019-0729-9 (PMC6421637; doi:10.1186/s12863-019-0729-9)

Additional file 1: Scatterplots of phosphorus content in starch of potato varieties analyzed, showing sensitivity of the third (A) and eighth (B) components to phosphorus content. The blue ellipses show varieties, which high or low phosphorous contents in starch are genetically determined.

A

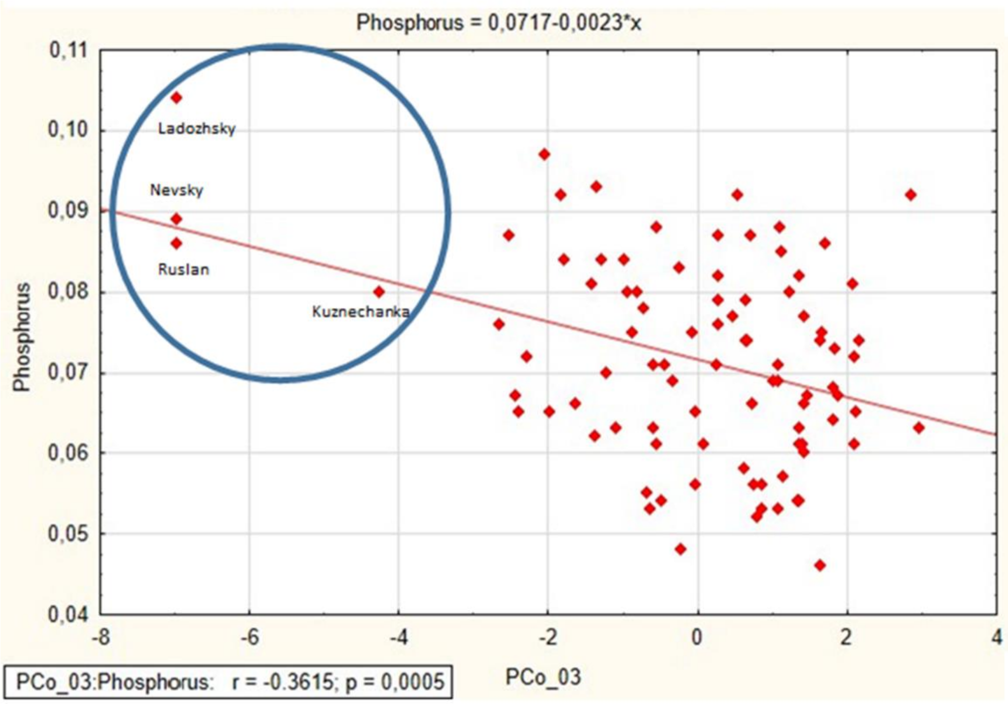

B

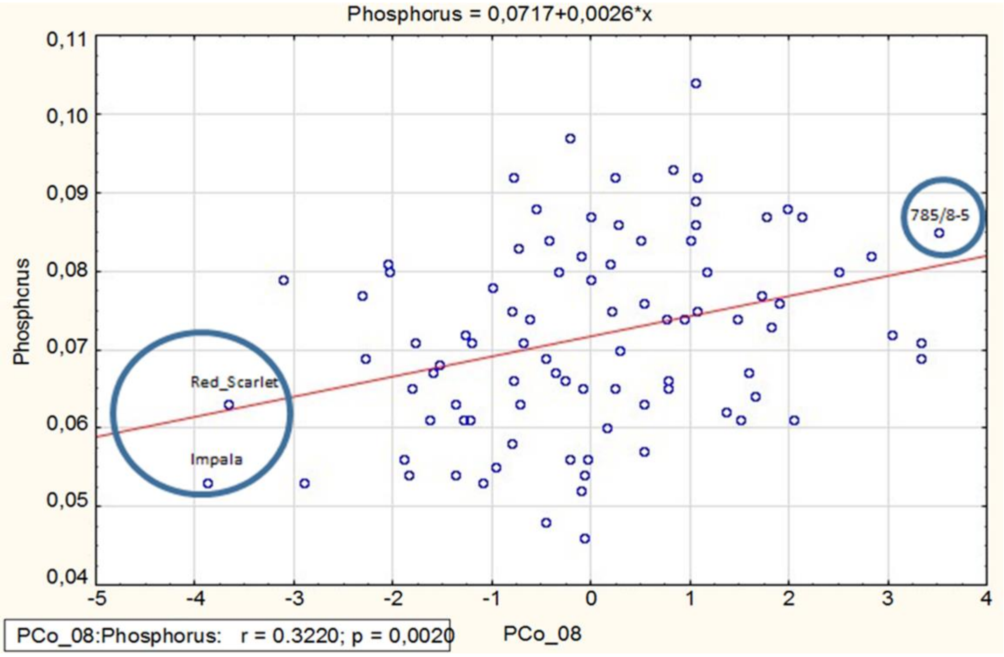

Supplement: Supplementary file 1 — Scatterplots of phosphorus content in starch of potato varieties analyzed, showing sensitivity of the third (A) and eighth (B) components to phosphorus content. The blue ellipses show varieties, which high or low phosphorous contents in starch are genetically determined. (PDF 314 kb) [file 12863_2019_729_MOESM1_ESM.pdf]
