# Supplementary material for: Starch phosphorylation associated SNPs found by genome-wide association studies in the potato (Solanum tuberosum L.)
Source: BMC Genet. 2019 Mar 18;20(Suppl 1):29. doi: 10.1186/s12863-019-0729-9 (PMC6421637; doi:10.1186/s12863-019-0729-9)
Supplement: Supplementary file 2 — QQ-plots (quantile-quantile plots) for the models: (1) GLM without correction for population structure; (2) GLM + Q: GLM + Q-matrix to account for population structure; (3) GLM + PCA, (4) MLM. (PDF 282 kb) [file 12863_2019_729_MOESM2_ESM.pdf]

**Additional file 2:** QQ-plots (quantile-quantile plots) for the models: (1) GLM without correction for population structure; (2) GLM + Q: GLM + Q-matrix to account for population structure; (3) GLM + PCA, (4) MLM.

**GLM**

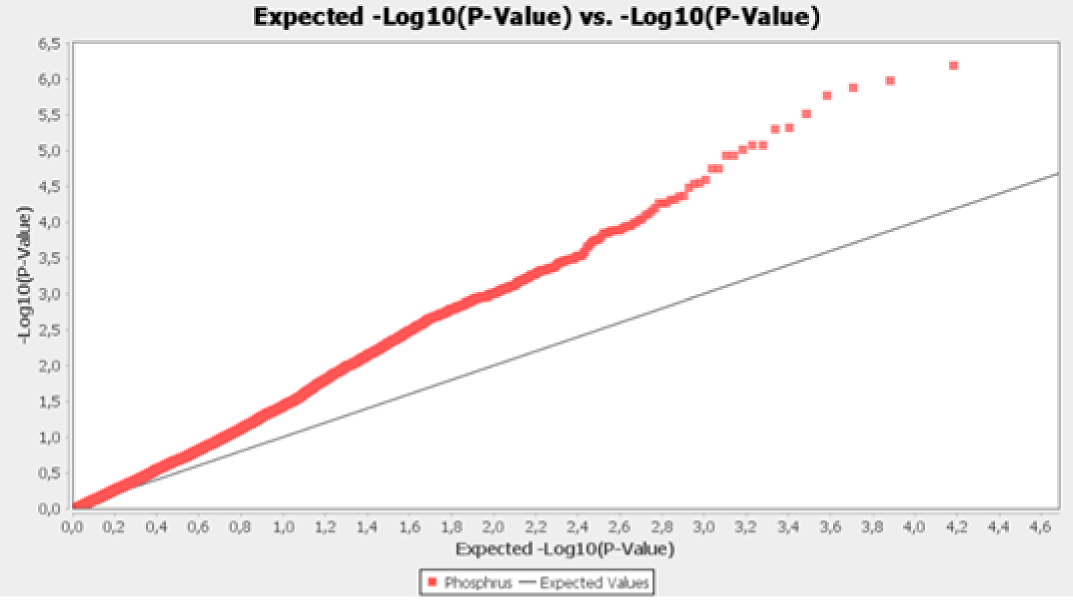


**GML+Q**

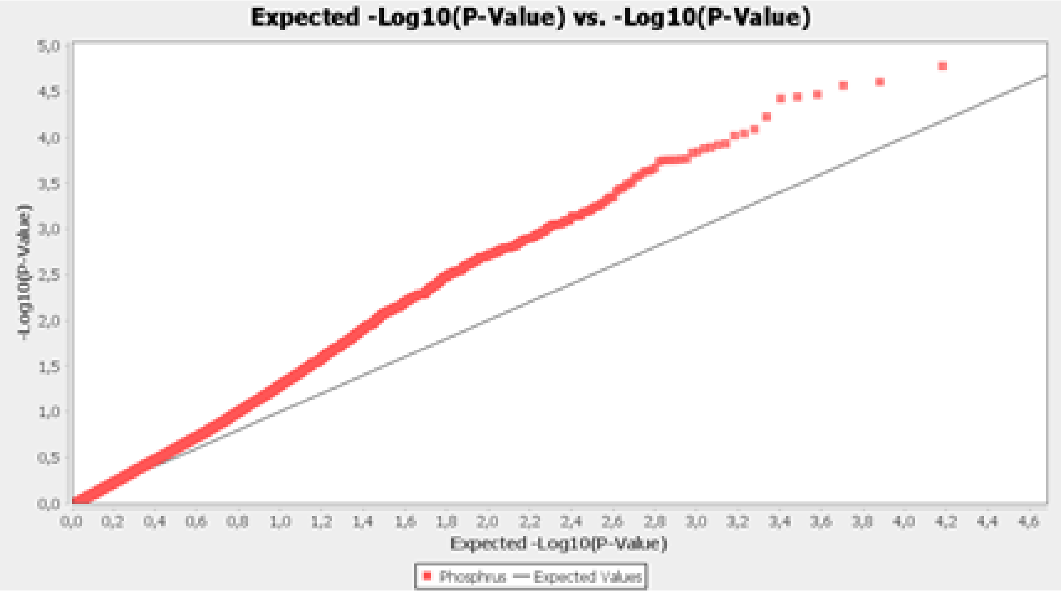

**GLM+PCA**

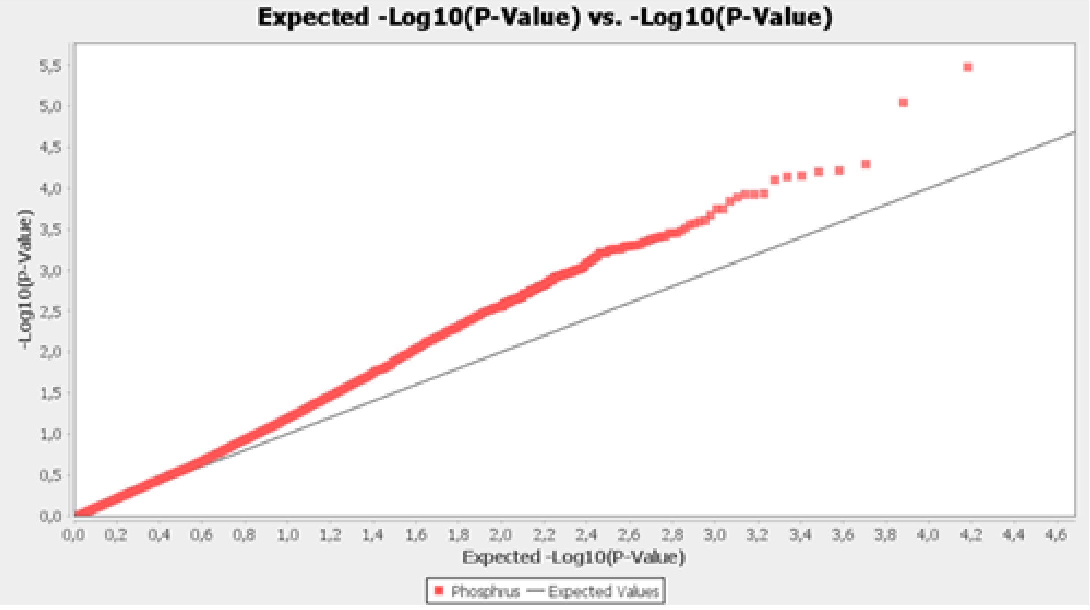
Expected -Log10(P-Value) vs. -Log10(P-Value)
-Log10(P-Value)
Expected -Log10(P-Value)
Phosphrus
Expected Values

**MLM**

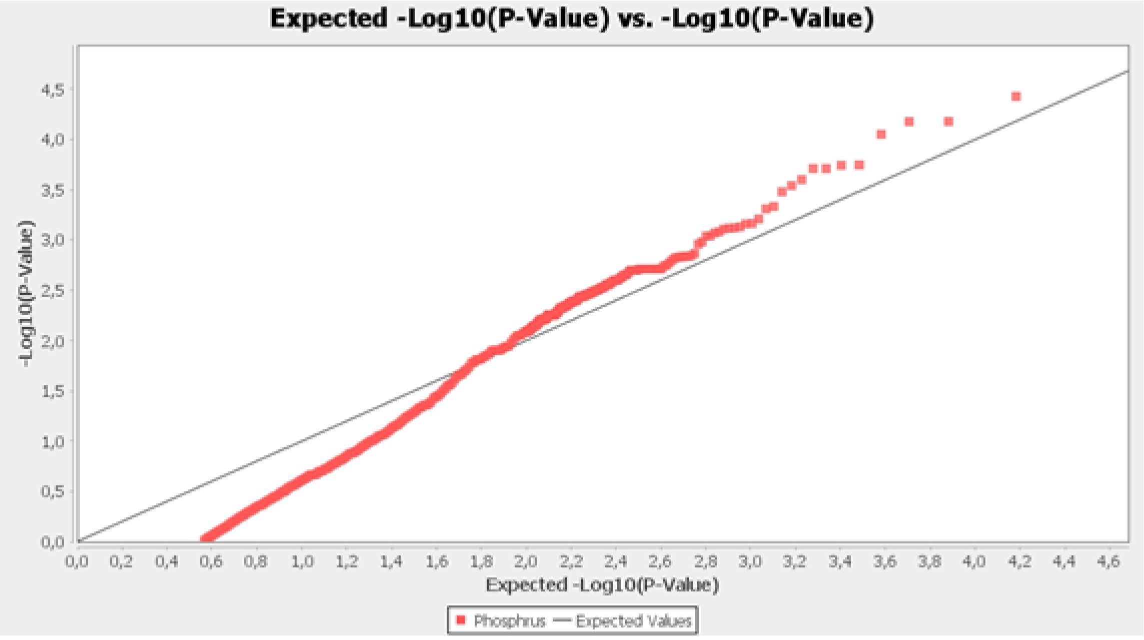
Expected -Log10(P-Value) vs. -Log10(P-Value)
-Log10(P-Value)
Expected -Log10(P-Value)
Phosphrus
Expected Values
