# Supplementary material for: Starch phosphorylation associated SNPs found by genome-wide association studies in the potato (Solanum tuberosum L.)
Source: BMC Genet. 2019 Mar 18;20(Suppl 1):29. doi: 10.1186/s12863-019-0729-9 (PMC6421637; doi:10.1186/s12863-019-0729-9)
Supplement: Supplementary file 3 — Association mapping results for phosphorous content scorings in potato starch using different models: GLM without accounting for population structure, GLM + Q (GLM + Q-matrix to account for population structure), GLM + PCA, and MLM. Dash line named “Bonferroni” corresponds the Bonferroni threshold. Dash line named “FDR” corresponds the FDR (false discovered rate) threshold. Vertical axes show the –log (10) of marker trait association p-value. Chr1–12 – chromosome assignment of loci. Chr0 – unassigned loci. (PDF 787 kb) [file 12863_2019_729_MOESM3_ESM.pdf]

Additional file 3: Association mapping results for phosphorous content scorings in potato starch using different models: GLM without accounting for population structure, GLM + Q (GLM + Q-matrix to account for population structure), GLM + PCA, and MLM. Dash line named "Bonferroni" corresponds the Bonferroni threshold. Dash line named "FDR" corresponds the FDR (false discovered rate) threshold. Vertical axes show the –log(10) of marker trait association p-value. Chr1-12 – chromosome assignment of loci. Chr0 – unassigned loci.

GLM without accounting for population structure

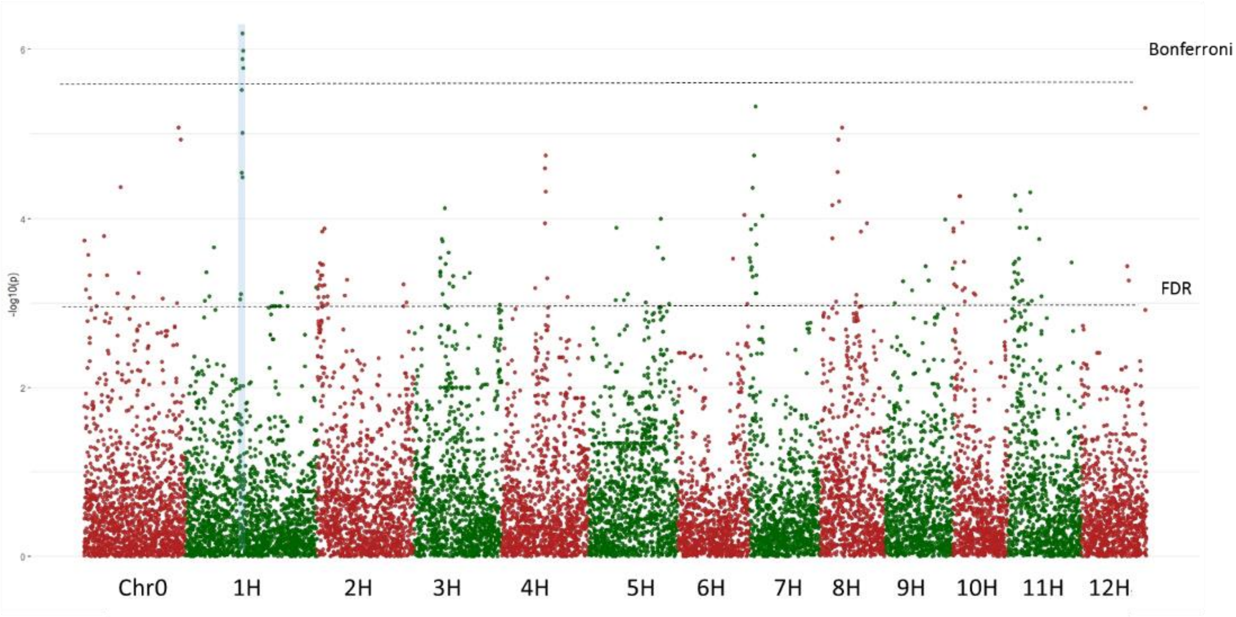

GLM + Q

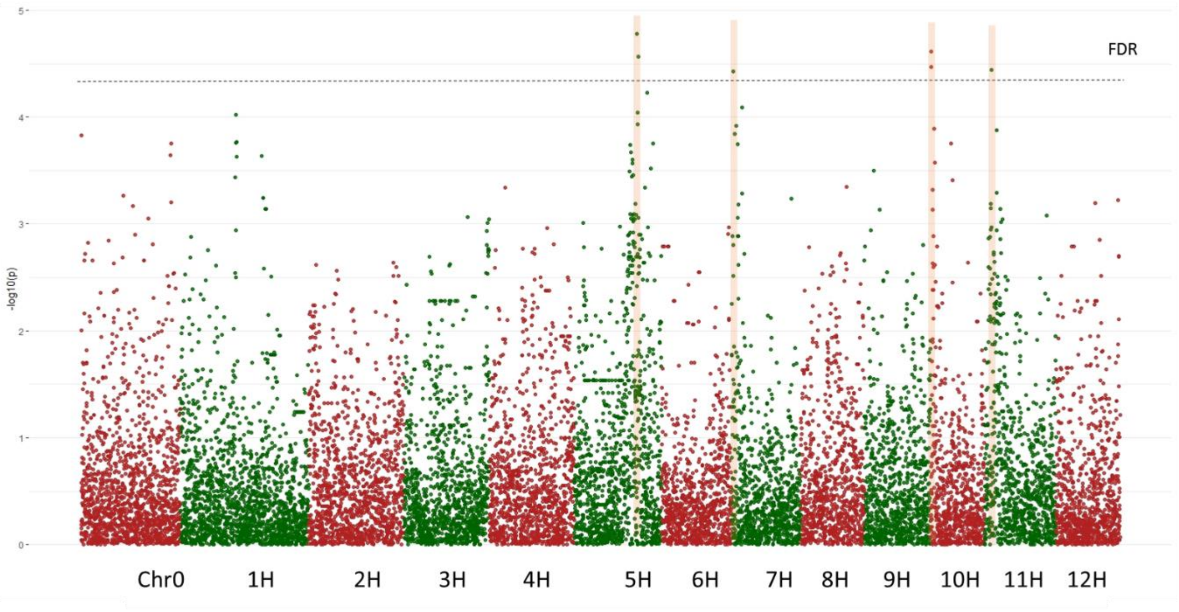

GLM + PCA

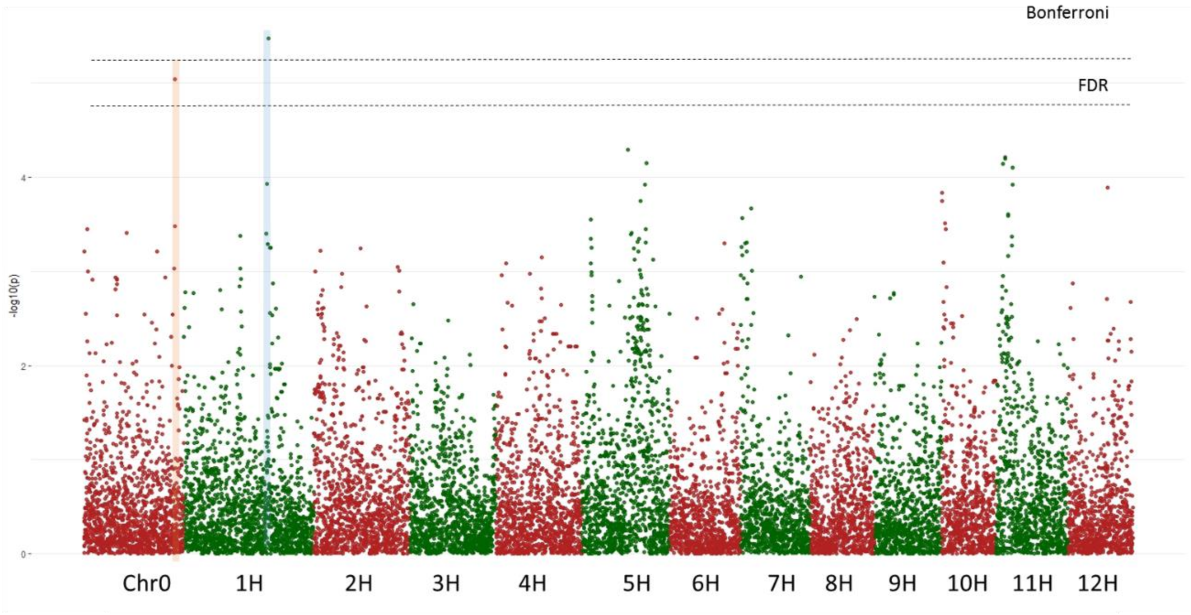
Bonferroni
FDR
-log10(p)
0
2
4
Chr0
1H
2H
3H
4H
5H
6H
7H
8H
9H
10H
11H
12H

MLM

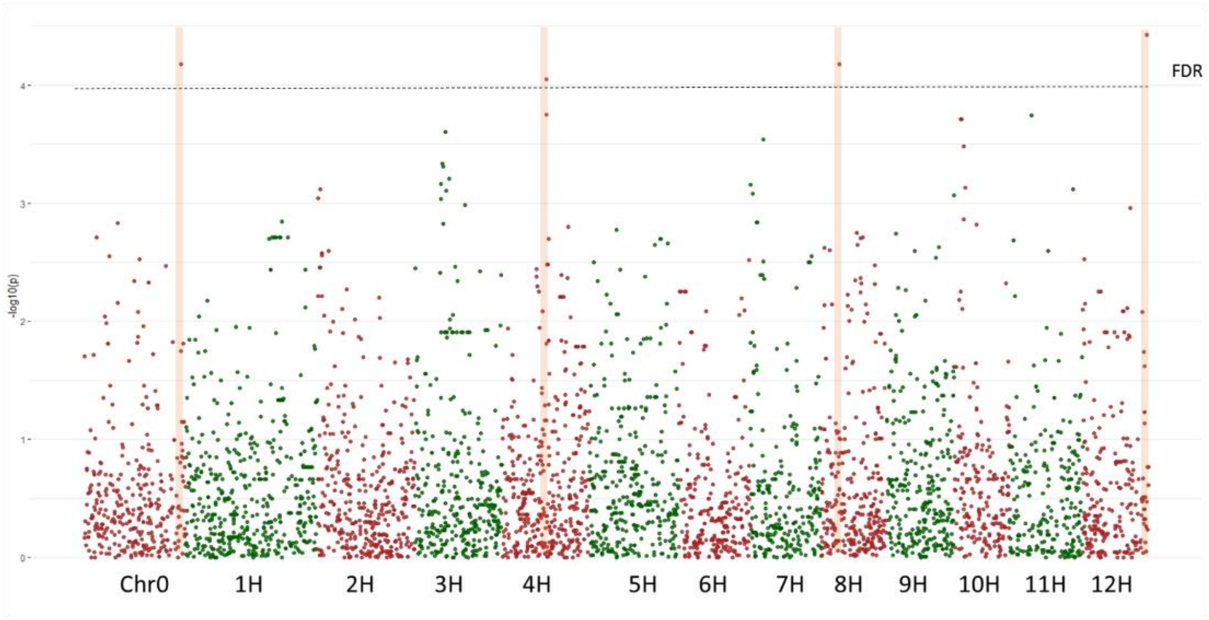
FDR
-log10(p)
0
1
2
3
4
Chr0
1H
2H
3H
4H
5H
6H
7H
8H
9H
10H
11H
12H
